# Economic analysis of reciprocating engine generating with bio-syngas at predicted maximum power condition

**DOI:** 10.1016/j.heliyon.2024.e34338

**Published:** 2024-07-11

**Authors:** Hiroshi Enomoto, Ryo Nakagawa, Ayako Yoshimichi

**Affiliations:** School of Mechanical Engineering, Kanazawa University, Kakuma-machi 1, Kanazawa, Ishikawa, 9201192, Japan

**Keywords:** Economic analysis, Bio-syngas, Reciprocating engine, Maximum power

## Abstract

In order to effectively utilize woody biomass, which has a low abundance density, it is necessary to develop a power generation system that can convert it with high efficiency even with a small capacity as less than 2 MW. For electricity generation, it is reasonable to use a small reciprocating engine. In the case of a naturally aspirated spark ignition reciprocating engine (SIRE), the amount of aspirated gas in one cycle is determined almost entirely by the displacement. The thermal efficiency of the SIRE generally increases with the power. Therefore, to improve the thermal efficiency, it is effective to make the low heating value (LHV) of the fuel higher to increase the power of the naturally aspirated SIRE. In this paper, three methods are used to increase the LHV of the bio-syngas: 1) reducing the nitrogen density of the bio-syngas (upgrade bio-syngas), 2) adding hydrogen to the bio-syngas, and 3) adding methane to the bio-syngas. Using these fuels, 1) the conditions for high power, and 2) the costs assumed for each condition, are evaluated through experiments and estimates. The results showed that the upgrade bio-syngas, obtained by gasification with oxygen-enriched air, had the highest power and the best cost-effectiveness.

## 1 Introduction

In recent years, the use of renewable energies has been promoted, and the IEA reported that biomass energy has high potential as an energy supply source on a global scale [1]. Since the estimated amount of sustainable biomass energy in 2050 is 200–500 EJ, there is enough potential for biomass energy demand in the same year. Therefore, Japan is desirable to use biomass energy effectively as an energy supply source.

**Fig. 1:**
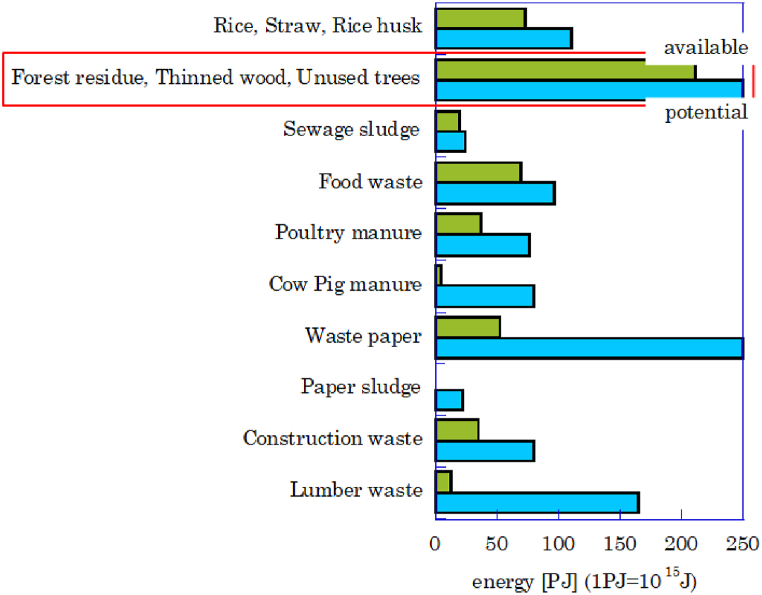
Biomass energy source in Japan [2].

**Fig. 2:**
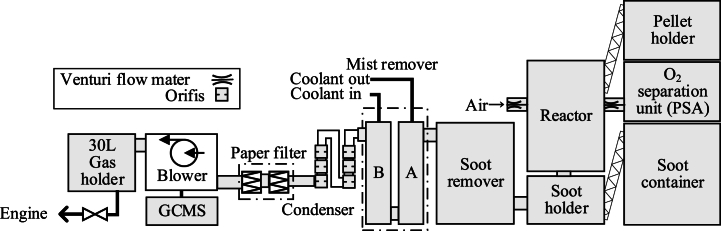
Schematic of gasification system.

Fig. 1 shows the biomass resources available in Japan by energy content [2]. The biomass potential and available are shown for each type. Waste biomass resources include mill residues and manure. Unutilized resources are wheat straw, while grass, algae, and vegetable oil are productive biomass resources. Fig. 1 shows that forest residues, thinned wood, unutilized trees, and recovered paper have the highest potential. Most of the recovered paper is already reused. On the other hand, forest residues, thinned wood and unutilized trees are not used, and therefore have the highest amount available. In order to utilize the unused biomass in Japan as electricity, it is necessary to define the specifications of a suitable power generation system (see Fig. 2).

In general, biomass is consumed for power generation 1) by direct combustion or 2) by gasification. An example of direct combustion is the power generation by steam turbines [3]. Gas turbines and gas spark ignition reciprocating engine (SIRE) are widely used for gasification. To select a biomass power generation system suitable for the climate and the topography of Japan, a country with a narrow tropical zone, the following two conditions must be confirmed. Firstly, Japan belongs to the tropics and is strongly affected by typhoons. As a result, natural disasters are frequent. Natural disasters interrupt the logistics. Therefore, the solution is to deploy power generation facilities with independent fuel supply sources in each region. Small-scale power generation systems are effective, and the gas SIRE and the gas turbines are suitable for this purpose. Secondly, Japan has narrow flatlands and many mountainous areas. As a result, a large-capacity power generation systems can only be installed in areas close to the coastline. Yoshioka et al. estimated that the transportation cost of woody biomass in Japan is higher than that in Europe [4]. They attributed this to 1) low forest density, 2) mountainous terrain, 3) lack of road network development, and 4) low penetration of efficient forestry machinery. Consequently, the large-capacity woody biomass power generation systems with steam turbines are not suitable for Japan's topography. From the viewpoint of fuel procurement cost, it is more appropriate to use a gas turbine or the gas SIRE for biomass power generation in narrow areas. In addition, a system with a general-purpose SIRE (GP-SIRE) as the engine for power generation is considered due to its availability and ease of maintenance. The GP-SIRE is easy to operate and is expected to make effective use of biomass resources [5].

### 1.1 Structure of a woody biomass gasifier suitable for a gas SIRE power generation

When woody biomass is used as fuel for the GP-SIRE, the fuel properties are liquid or gas. Bridgwater et al. calculated the product rate extracted for each pyrolysis condition [6]. They reported that 75 % of the calorific value of the input woody biomass can be extracted as liquid in the fast pyrolysis condition. However, authors consider that further modification is necessary as fuel for the engine because it contains water a lot. On the other hand, when woody biomass is extracted as gas instead of liquid, the cold gas efficiency (CGE) is 82.7 % (averaged) [7]. For a power generation, where conversion efficiency is an important index, converting woody biomass to gas is more appropriate. Therefore, the use of a SIRE is rational for this gaseous fuel. In this paper, the combustible gas obtained from the gasification of woody biomass is defined as the bio-syngas.

The gasifier in a small-capacity power generation system has a small reaction zone, so the appropriate reaction system must be selected for this gasifier. The reaction zone of a gasifier can be classified into the following categories according to the state of the fuel during the reaction: 1) fixed-bed type, 2) moving-bed type, 3) fluidized-bed type. All these types are self-heating because of their small capacities. In the fixed-bed type, the fuel's position is fixed by a grate in the reactor. In the moving-bed type, the fuel is transported in the reactor by a mechanical structure. In the fluidized bed type, the oxidizer flow conveys the fuel. Among these reaction systems, the fixed-bed type has the advantage of a small size and low manufacturing cost [8]. Therefore, fixed-bed gasifiers are suitable for small-capacity power generation systems.

The fixed-bed gasifiers emit tar along with the product gas. Tar is defined as "organic matter that is generally almost all aromatic and is produced during the pyrolysis or gasification of organic matter." [9]. Tar is highly viscous at room temperature and can cause blockages and failures in equipment. The tar concentration of 10 mg/Nm^3^ to 100 mg/N m^3^ is recommended for the bio-syngas supplied to the small SIRE to prevent mechanical structure failure [9].

The fixed-bed gasifiers are classified into downdraft and updraft types, each with a different oxidizer flow direction. The updraft type allows the oxidizer to flow from the bottom in relation to the fuel, while the downdraft type allows the oxidizer to flow from the top. The advantage of the downdraft type is the less tar emissions [8,10]. Therefore, a fixed-bed downdraft gasifier is suitable for producing the fuel for the small SIRE, and this system is used in this paper.

Syngas is a synthesis gas with a CO/H_2_ mixture, a ratio of CO and H_2_ of 1:1 in volume [11]. On the other hand, the bio-syngas, which is produced from woody biomass reacted by self-exothermic heating, is a combustible gas by the gasification with air as an oxidant. Therefore, the bio-syngas is a mixture of CO and H_2_ as the main combustible materials, with N_2_ as the inert component (>50 %) and CO_2_ (>10 %). As a result, the low heating value (LHV, [MJ/Nm^3^-LHV]) of the bio-syngas is from 4.5 MJ/m^3^-LHV to 5.6 MJ/m^3^-LHV [7,12,13], which is almost 1/10 of that of conventional fossil fuels [5]. Digestion gas is one of the combustible gases produced by methane fermentation of organic matter such as sewage sludge (biomass) and is composed mainly of CH_4_ and CO_2_. The methane content of digester gas is about 50 %–70 % [14], and its LHV is almost half of conventional fossil fuels. Compared to digestion gas, the inert gas fraction in the bio-syngas is large and the LHV is much smaller.

### 1.2 Need for the LHV adjustment

Przybyła et al. investigated the relation between the LHV of synthesis gas and the indicated mean effective pressure **(**IMEP) and the covariant of indicated mean effective pressure (COV-IMEP) of the GP-SIRE [15]. They used mixtures containing CO (44v%), H_2_ (17.4v%), and N_2_ (38.6v%) diluted with N_2_ to supply eight different LHVs (from 2.94 MJ/m^3^-LHV to 7.44 MJ/m^3^-LHV). The maximum IMEP decreased with decreasing LHV. The COV-IMEP was largest at LHV of 2.94 MJ/m^3^, reaching 4.9 %. From the above, the LHV affects the IMEP and the COV-IMEP.

Since wood biomass power generation systems are usually stationary, the temperature of the oxidizer varies from season to season. In Suzu City, Ishikawa Prefecture, the minimum average temperature in winter is −0.8 °C and the maximum average temperature is 30.3 °C in summer. The temperature difference between winter and summer changes the oxidizer density by almost 15 %. Consequently, the amount of combustible gas aspirated by the SIRE changes, and the power varies with the seasons. Normally, the SIRE for power generation is used throughout the year, so they must have a power adjusting function to cope with this seasonal change. On the other hand, the SIRE has a short response time for power change, so it is necessary to change their operating conditions in response to power demand change. Generally, when the SIRE responds to the seasonal demand change (power change), the engine speed is varied. However, in case of the power generation, the engine speed changes extremely, the alternator is heavily loaded and the electricity quality is results poor. Therefore, a rational method of responding to this power change is to change the LHV of the bio-syngas. Effective methods to change the LHV are 1) the addition of CH_4_ or H_2_, which has a low environmental impact, and 2) changing the oxidizer for gasification. In this paper, the bio-syngas with H_2_ is defined as the bio-syngas added with H_2_, and the bio-syngas with CH_4_ is defined as the bio-syngas added with CH_4_. The oxidizer for gasification is oxygen-enriched air (a mixture of oxygen and nitrogen with a higher percentage of oxygen than air; the O_2_ concentration of the oxygen-enriched air obtained by the equipment in this experiment is not 100%-O_2_. Then, it is defined as O_2-quasi_ by adding “-quasi” after the O_2_ notation).

There are three advantages of the oxygen-enriched air to produce the upgrade bio-syngas. Firstly, since N_2_ in air is an inert gas, a decrease of the N_2_ fraction in the bio-syngas increases the LHV [16]. When the LHV increase, the power of the power generation system become large, thus the unit cost of the system decrease. Second, in the case of increasing the O_2_ concentration in the oxidant, the gasification rate of the woody biomass become fast, and the size of the reaction zone can be reduced. The increase of the O_2_ concentration in the oxidant causes the rise of the reaction field temperature [16] and the chemical reaction rate. It is explained by the Arrhenius-type chemical reaction equation. Third, the increase in the reaction field temperature promotes the thermal decomposition of tar. Therefore, tar emissions can be reduced [17].

The higher the power of the SIRE, the more valuable it is as a power generator. Therefore, when syngas is supplied to the SIRE, the fuel composition that produces higher power should be selected. For a naturally aspirated SIRE, the engine power is maximum when the charging efficiency is maximum. The IMEP is an index of the power of the SIRE, but it is different from the commonly applied torque [Nm] or power [kW]. The definition of the IMEP is “the value which divide an indicated work by the displacement volume”, and the difference of power due to differences of displacement can be ignored. For example, Negurescu et al. showed that at maximum charging efficiency, the IMEP is 1.0 MPa when the engine is operating with gasoline and 1.3 MPa operating with H_2_ [18]. Przybyła et al. also studied the maximum IMEP for the LHV of the bio-syngas [15], and Ando et al. did the same study. However, these previous studies do not allow us to discuss the superiority or characteristics of the maximum power of engines operating with three different bio-syngas mixtures enough. When the composition of the fuels is different, even if the LHV of the fuel and the air excess ratio are equal, the stoichiometric amount of air also changes, and the heat supplied at maximum charging efficiency differs. Therefore, the maximum power of the GP-SIRE operating with the three different bio-syngas mixtures is estimated by the IMEP and the maximum charging efficiency in this paper. The IMEP under maximum power operating conditions is defined in the Results and Discussion section. The fuel flow rate is adjusted to keep the excess air ratio (EAR) constant.

The objective of this paper is to select the most cost-effective gas among the three bio-syngas mixtures under the maximum power operating conditions. For this purpose, the flow rates of the oxidizer O_2-quasi_ to produce the upgrade bio-syngas, the bio-syngas containing CH_4_, and the bio-syngas containing H_2_ are predicted and their respective prices are calculated.

## 2 Experimental apparatus

### 2.1 Gasifier

A self-made gasifier is used for the experiment. Whole cedar pellets, which are readily available, are for the fuel. The pellets are 6 mm in diameter, 15 mm long, and have a moisture content of 8.0w%. The pellets are fed from a pellet holder to the reactor by a screw. The oxidizer for the fuel is either air or air + O_2-quasi_. The O_2-quasi_ is a mixture of oxygen and nitrogen separated from air by the oxygen concentrator (maximum flow rate 10 L/min, O_2_ concentration 90v% ± 3). Since the O_2_ concentration of the oxygen-enriched air produced by this apparatus is not 100 %, “-quasi” is added after the O_2_ notation. The O_2_ concentration measured by gas chromatography (Agilent Technologies 490 Micro GC) is approximately 87v%. The flow rate of the oxidizer was measured by a self-made Venturi flowmeter. A blower installed downstream of the gasifier maintains a negative pressure inside the reactor and creates a flow from upstream to downstream in the reaction zone.

**Fig. 3:**
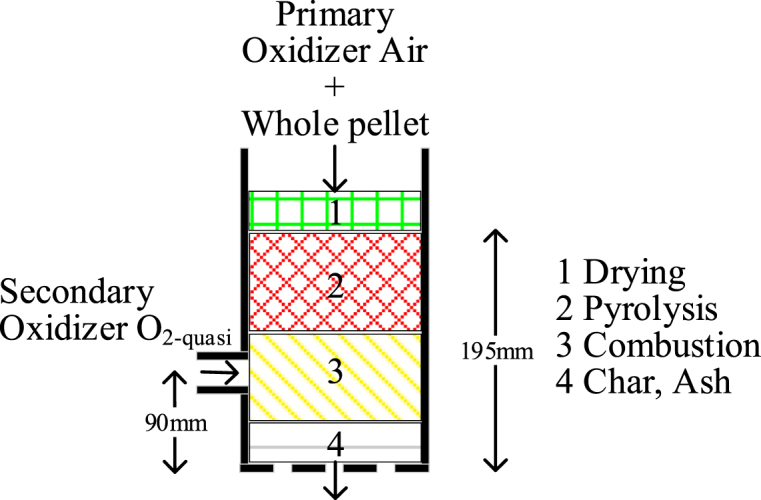
Oxidizer and pellet flow inside of reactor.

Fig. 3 shows the concept of the reactor. The inside diameter of the reactor is 102.3 mm. A level installed in the reactor detects a decrease in pellet height. The pellets maintain the height of 195 mm by the detection signals activate the pellet feed. A grate fixed inside the reactor supports the pellets. If O_2-quasi_ is used as the oxidizer, it is fed at 90 mm from the bottom of the pellets; it can also be mixed with a primary oxidizer and fed from the top of the reactor. However, this method results in an excessively high gasification reaction rate of the pellets, and the pellets are reduced to ash in a short time. It causes the reactor block easily. Therefore, O_2-quasi_ is fed at the 90 mm position in this paper.

The bio-syngas composition produced in the gasifier is measured by a gas chromatography (Agilent Technologies 490 Micro GC). The bio-syngas is removed by a moisture removal system. The columns for the gas composition analysis of H_2_, N_2_, O_2_, CH_4_, and CO are Molsieve 5A 10 m and PoraPLOT Q 10 m.

When woody biomass is gasified, solid soot, water vapor and tar are produced simultaneously with the bio-syngas. For soot removal, recycled glass gravel with a diameter of approximately 20–30 mm was placed to separate the gas and solid. The mist remover is water-cooled to lower the temperature of the gaseous component and remove water vapor and tar as a drain. A condenser installed in the backstream of the mist remover takes out water vapor and tar as the drain by the temperature decrease due to adiabatic expansion. The soot removal system downstream of the reactor itself was not sufficient to separate solids. Therefore, a paper filter installed downstream of the condenser removes soot again.

**Fig. 4:**
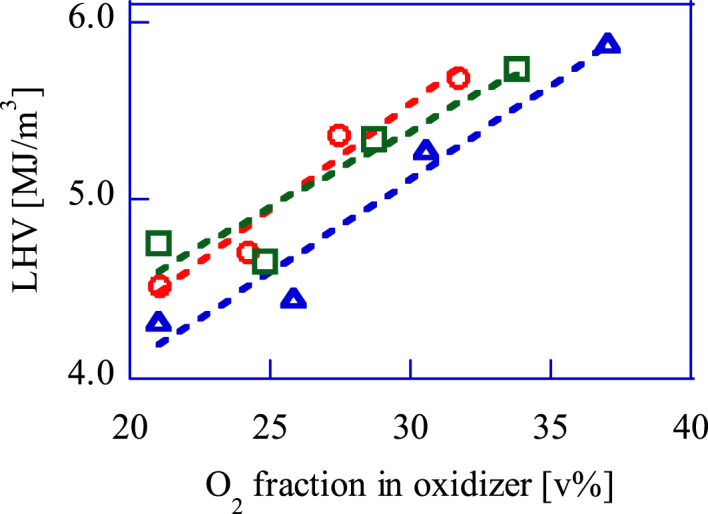
The upgrade bio-syngas LHV change by O_2_ fraction control in oxidizer. Total oxidizer flow rate, 〇: 60 L/min, □ 50 L/min, △ 40 L/min.

Fig. 4 shows the relation between the O_2_ volume fraction in the oxidant and the LHV. The LHV of the bio-syngas changes as the O_2_ volume fraction in the oxidant changes. The air flow rate was adjusted so that the total oxidizer flow rate was 40, 50, and 60 L/min under the conditions of 0, 3, 6, and 10 L/min for the O_2-quasi_ flow rate. When the total oxidizer flow rate (mixture of air and O_2-quasi_ air) is 60 L/min, the reactor temperature is the highest and the gasification reaction rate of the pellets increases. Therefore, the LHV increased as the total oxidizer flow rate increased. Comparing conditions with 21 v% and 25 v% O_2_ volume fractions in the oxidizer, the LHV hardly changes. This means that the LHV of the bio-syngas can be significantly modified under conditions where the O_2_ volume fraction in the oxidizer exceeds 25 v%.

### 2.2 GP-SIRE

**Fig. 5:**
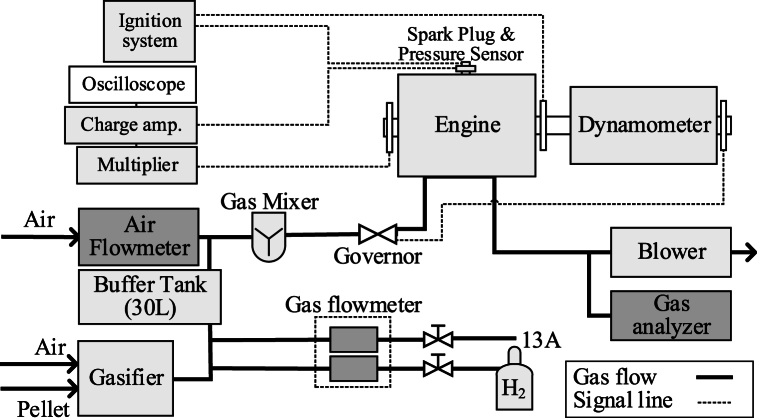
Schematic of SI-ICE experimental equipment.

**Fig. 6:**
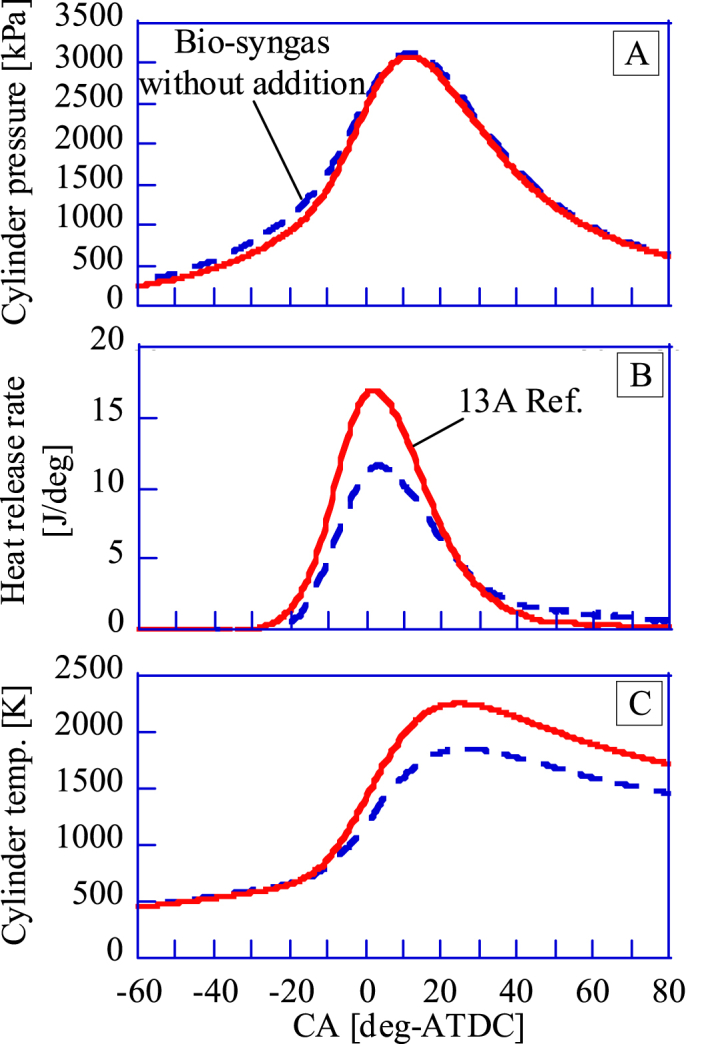
Time histories of cylinder pressure and derivatives.

Table 1 shows the engine specification in the experiment. A general-purpose V-twin cylinder engine was converted to a single-cylinder drive to prevent variations in the intake air flow mixture for each cylinder. Remove the pushrods that operate the intake and exhaust valves to prevent one cylinder from doing the work. Fig. 5 shows a schematic diagram of the experimental setup for the engine performance tests. Typical time histories are shown in Fig. 6 (0.6 MPa-IMEP). This figure shows the comparison between normal bio-syngas and city gas (13A).

**Table 1:** Specifications of the SIRE.

Manufacturer	Kawasaki Heavy Industries, Ltd.
Model	FD750D
Engine type	OHV, 90°V-twin, Liquid-cooled
Combustion chamber type	Pent-roof, 2 valves
Displacement [cm3]	745 → 372.5 modified
Number of cylinders	2
Bore x Stroke [mm]	78 x 78
Length of con-rod [mm]	130
Piston offset [mm]	1
Compression Ratio	8.6 : 1
Rated power [kW]	18.7 (at 3600 rpm)
Rated torque [Nm]	55.6 (at 2600 rpm)
Valve timing (measured)	IVO: 48 deg-BTDC
	IVC: 42 deg-ABDC
	EVO: 44 deg-BBDC
	EVC: 8 deg-BTDC

H_2_ is supplied from a cylinder to add H_2_ to the fuel. City gas (13A) is supplied as an alternative to add CH_4_. These gas flow rates are adjusted by needle valves. The flow rate of the added fuel was measured by a thermal mass flow meter (azbil CMS0050). The bio-syngas and H_2_ or CH_4_, produced in the gasifier, flow from each fuel feed line to a 30 L buffer tank.

The intake air flow rate of the GP-SIRE is measured by a laminar flowmeter (Sokken LFE-25B) and is changed by electronic governor (Woodward LC-50). The governor controls the valve opening with PID to maintain a constant engine speed for the GP-SIRE. This electronic governor operates by PID control of the governor opening to maintain a constant engine speed for the GP-SIRE. Intake air and the fuel which passed through the buffer tank are supplied to the GP-SIRE through a gas mixer.

An eddy current dynamometer (EWS-150-L, Tokyo Meter Co., Ltd.) is to measure the power and speed of the GP-SIRE. The load of the dynamometer is controlled by an automatic dynamometer controller (EDC-280-ZX, Tokyo Meter Co., Ltd.).

For a spark plug and in-cylinder pressure sensor of the GP-SIRE, 6118CF-5CQ03S4-2 by KISTLER is used. Ignition timing is controlled by an ignition timing controller (Altronic, CD200D). In-cylinder pressure of the GP-SIRE is recorded by a combination of an in-cylinder pressure sensor and a charge amplifier (5018A) by KISTLER. For the data analysis, a memory recorder (DL850) by Yokogawa Electric Corporation and engine combustion analysis software (720340) are used. For the memory recorder, a charge amplifier, and a multiplier for crank angle measurement (Atosense Corporation, CPM-100) are connected to the memory recorder. The in-cylinder temperature is calculated based on the equation of state, assuming complete combustion of the fuel (see supplement). Therefore, the validity should be examined. Fig. 6 shows the measured A: pressure, B: heat generation rate, and C: in-cylinder temperature. The estimated in-cylinder temperature in this figure is almost the same as the in-cylinder temperature obtained by Kan et al. in a CFD simulation by KIVA4 [19]. Therefore, the calculations in this paper are appropriate.

A commercial radiator (FD750D) is for cooling the GP-SIRE. A flow meter (TOFCO, HF-GCT40-01-30-04) is for measuring the cooling water flow rate. Cooling water is circulated by an electromagnetic pump, and the temperature is maintained the inlet cooling water at a constant range of 80 ± 2 °C by the circulator.

For measuring the volume fraction (NOx, HC, CO, CO_2_) in the exhaust gas of the GP-SIRE, HORIBA MEXA-584L are used. Moisture in the exhaust gas is removed by a drain separator.

## 3 Experimental conditions

The bio-syngas or the upgrade bio-syngas produced in the gasifier input to a Kawasaki Heavy Industries FD750D converted to a single-cylinder drive. The fuel flow rate is adjusted to an EAR of 1.6 ± 0.1. When the power of the GP-SIRE is equal, the fuel input decreases as the EAR increases, so the indicated thermal efficiency increases. When CH_4_ is supplied to the GP-SIRE, the EAR should be 1.0 ± 0.1. Preliminary experiments show the indicated thermal efficiency is generally maximum when the EAR is 1.6 ± 0.1 or higher in the case of the bio-syngas included gas fuel. The EAR for CH_4_ operation is different from that for operating with the bio-syngas to prevent engine stalls that frequently occur in the fuel lean region when operating with CH_4_.

**Fig. 7:**
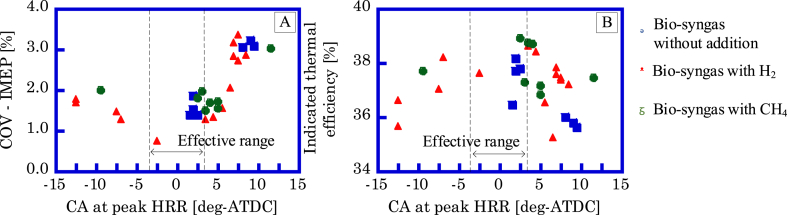
A:COV-IMEP [%] and B:indicated thermal efficiency for CA at peak HRR.

The range of ignition timing for the GP-SIRE is set to ±3.0 deg-ATDC for the crank angle (CA, [deg-ATDC]) at which the heat release rate (HRR, [J/deg]) is maximum. Fig. 7A shows the relation between the COV-IMEP and the CA at which HRR is maximized. Fig. 7B shows the relationship between the indicated efficiency and the CA at which the HRR is maximized. When the HRR is maximum before the compression top dead center (TDC), most of the fuel generates heat during the compression process. The pressure rise in the compression process is negative work and leads to losses and combustion instability. When the HRR is maximum after the TDC, most of the heat is generated in the expansion process. Since the degree of constant volume in the expansion process is small, the indicated work is reduced and leads to losses and combustion instability. When the cylinder's volume change is smallest to the CA change, the CA is near TDC. Consequently, when the heat generation of the fuel is maximum at the CA with high isobaric CA, the indicated work is the maximum and the combustion is highly stable. The detailed ignition timing setting method for the GP-SIRE operating with the bio-syngas is explained by Enomoto et al. [20].

The engine speed of the GP-SIER should be 1800 ± 22.5 rpm for a power generator. JIS B 8009-5 provides standards for a single-cylinder or a two-cylinder GP-SIRE as a power generator use. This standard determines the generator speed variation by the steady-state frequency change rate. The allowable rate of change is determined as 2.5 % for the same use as the commercial power supply with the voltage specification. Therefore, this value is used.

## 4 Results and discussion

### 4.1 Definition of maximum power conditions

In this paper, the maximum power is expressed by the IMEP. The calculation method of the IMEP is shown in the Supplement. In general, the engine power is maximum when the charging efficiency of a naturally aspirated SIRE is at maximum. Based on this fact, the maximum power condition for the GP-SIRE operating with the bio-syngas is defined. In this paper, the reference values for evaluating the power of the GP-SIRE operating with the bio-syngas are obtained by measuring the charging efficiency and the IMEP operated with CH_4_. The maximum power of the engine in the experiment can be estimated from catalog value. The catalog lists the gross shaft torque, which is a shaft torque measured with only the auxiliary equipment necessary for engine operation. The maximum gross shaft torque of the GP-SIRE is 52 Nm at 1800 rpm when CH_4_ is for the fuel. In this experiment, 1) intake components, 2) fuel, and 3) number of cylinders are changed from the stock engine. Therefore, the maximum gross shaft torque obtained at 1800 rpm is expected to be 23.4 Nm. In preliminary experiment, the shaft torque was 23.1 Nm and the charging efficiency was 0.89. Therefore, the maximum charging efficiency operating with CH_4_ is determined to be 0.90. The IMEP was 0.90 MPa in this experiment. The engine power is maximum at the maximum charging efficiency when the LHV of the fuel such as CH_4_ is high. However, in the case of the bio-syngas operation with low LHV, the engine speed tends to be unstable and the engine stalls easily under the higher power operation condition with higher charging efficiency. Therefore, the maximum power condition should be represented by a different index of charging efficiency when the bio-syngas is included in the fuel.

**Fig. 8:**
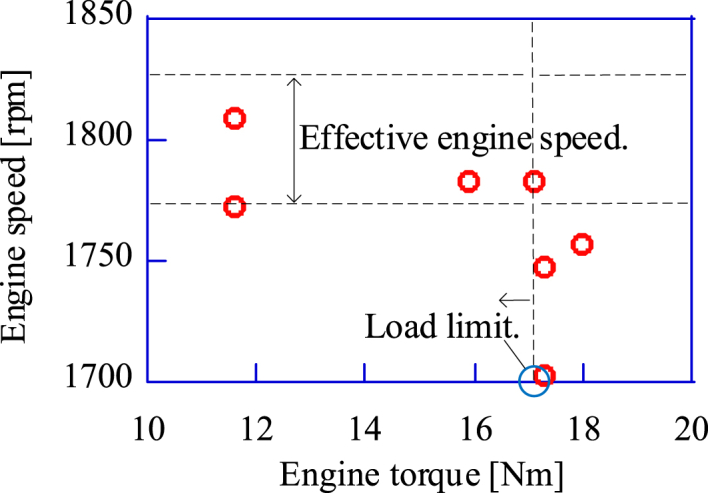
Engine speed change against increasing torque.

Under the maximum power conditions, the engine speed often becomes unstable. When the instability occurs under the maximum power conditions, the average RPM drops (Fig. 8). The EAR is 1.6 ± 0.1 and the upgrade bio-syngas (6.6 ± 0.1 MJ/m^3^-LHV) is as fuel. The engine speed decreases to 1700 rpm when the torque is around 17 Nm. At this time, the RPM fluctuation was about 2 %, and extremely unstable combustion was not observed (“back-fire” is not occurred). This is a phenomenon that has not been observed with conventional fossil fuels, but a detailed explanation is required and will be presented in the next paper. When the engine speed decreases to 1700 rpm, the charging efficiency is 0.84 due to insufficient heat input, which does not reach the maximum value operating with CH_4_. Therefore, the maximum power of the GP-SIRE operating with the three different bio-syngas mixtures are estimated by the IMEP as the maximum power at which the engine speed can be maintained at 1800 ± 22.5 rpm.

### 4.2 Maximum power operating with the upgrade bio-syngas and the required N_2_ ratio in the fuel

**Fig. 9:**
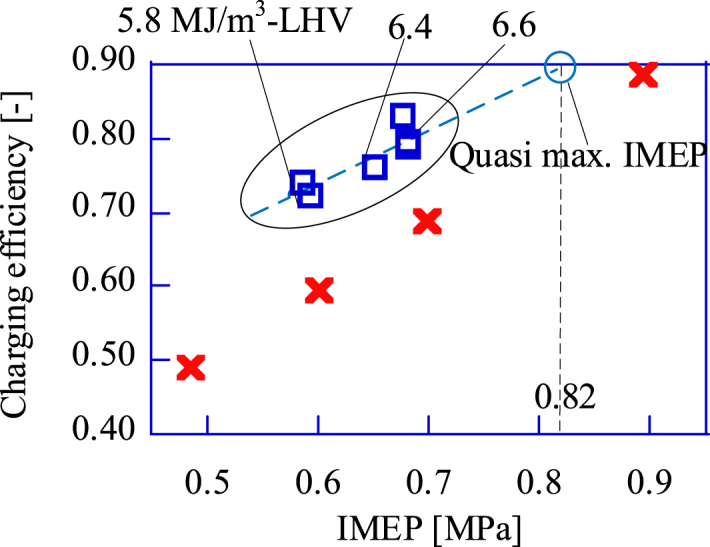
Calculated value of charging efficiency against measured IMEP. □:Upgrade bio-syngas, × :CH_4_(Ref.).

The maximum power that the GP-SIRE operating with the upgrade bio-syngas can achieve is predicted by the several IMEP results. Fig. 9 shows the relation between the IMEP and charging efficiency when operating with the upgrade bio-syngas and CH_4_. The gasifier in this paper was designed to vary the composition of the upgrade bio-syngas by adjusting the oxygen concentration of the oxidizer supplied to the gasifier. As a result, the maximum LHV is 6.6 MJ/m^3^, and the charging efficiency is 0.80 at this LHV. Therefore, as shown in Fig. 9, the IMEP at the maximum charging efficiency of 0.9 should be predicted to calculate the cost effectiveness. From a linear approximation in Fig. 9, the predicted IMEP, at which the charging efficiency is 0.9, is 0.82 with the upgrade bio-syngas.

**Fig. 10:**
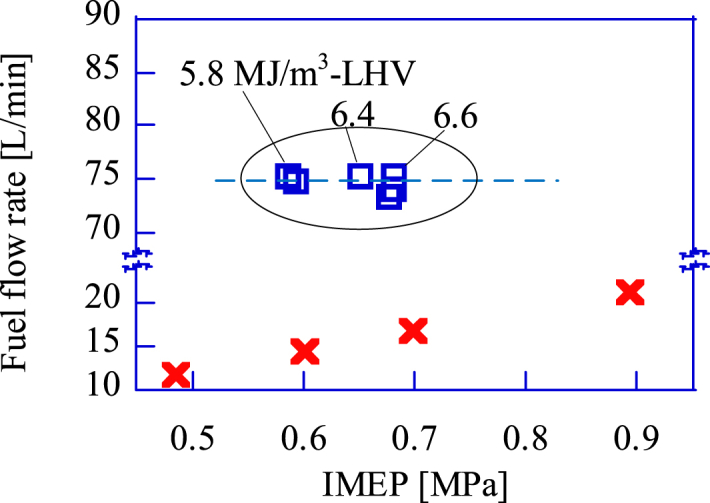
The fuel flow rate change for increasing IMEP. □:Upgrade bio-syngas, × :CH_4_(Ref.).

**Fig. 11:**
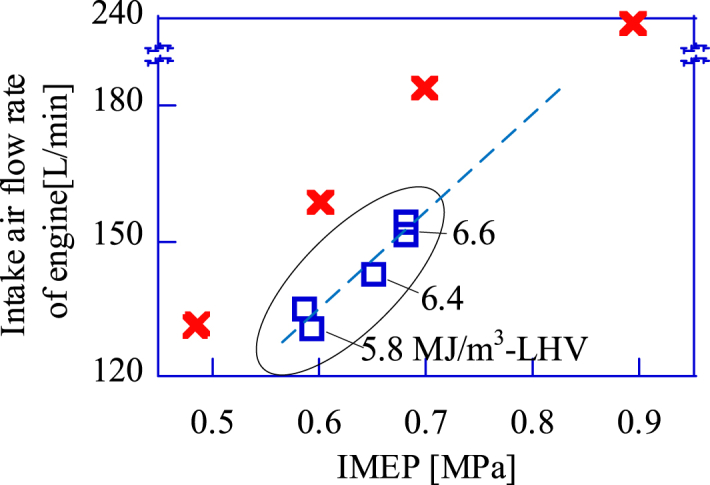
The intake air flow rate change for increasing IMEP. □:Upgrade bio-syngas, × :CH_4_(Ref.).

Fig. 10 shows the relation between the IMEP and the fuel flow rate supplied to the GP-SIRE. The indicated work of the GP-SIRE is proportional to the heat input when the thermal efficiency is constant. For CH_4_ operation, the LHV is constant. Therefore, for a larger IMEP, increasing the heat input is equivalent to increasing the fuel flow rate. On the other hand, in the case of the upgrade bio-syngas operation, the nitrogen fraction of the upgrade bio-syngas should be reduced to increase the LHV, since the EAR is constant. In this case, the fuel flow rate does not change as the IMEP increases. On the other hand, the engine intake air flow increases linearly with the increase in LHV of the upgrade bio-syngas (Fig. 11). This linearity confirms the linear relation between the charging efficiency and the IMEP for varying the LHV in Fig. 9.

**Fig. 12:**
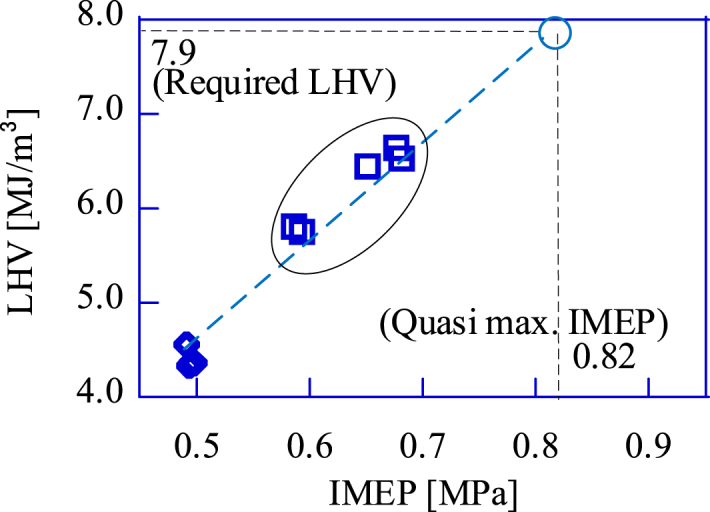
The maximum power (IMEP [MPa]) vs. the LHV [MJ/m^3^] for the upgrade bio-syngas. □:Upgrade bio-syngas, ◇:Bio-syngas without addition.

Fig. 12 shows the relation between the LHV and the maximum IMEP for the upgrade bio-syngas and the bio-syngas without addition. Since the maximum indicated work for the SIRE is proportional to the heat input, there is a linear relation between the IMEP and the LHV. Similar results were reported in an experiment conducted by Przybyła et al. [15]. As the maximum IMEP of the GP-SIRE operating with the upgrade bio-syngas is 0.82 MPa, the required LHV is 7.9 MJ/m^3^-LHV. The N_2_ fraction required to produce the upgrade bio-syngas at this LHV can be predicted from our experimental results. The LHV of combustible gas produced by the gasifier in this paper will not fall below 4.5 MJ/m^3^-LHV unless a system failure occurs.

**Fig. 13:**
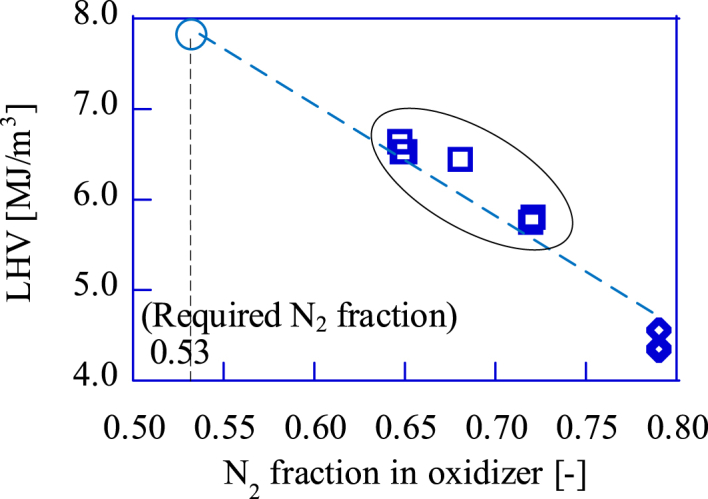
The effect of N_2_ volume fraction in oxidizer on the upgrade bio-syngas LHV. □:Upgrade bio-syngas, ◇:Bio-syngas without addition.

Fig. 13 shows the relation between the LHV and the N_2_ volume fraction in the oxidizer. The maximum LHV of the upgrade bio-syngas which produced in the experiment depends on the lower limit of the N_2_ fraction of the oxidizer. The 6.6 MJ/m^3^, the bio-syngas LHV shown in Fig. 13, is the maximum value that could be achieved with the experimental apparatus in this paper. The relation between LHV and N_2_ concentration in the oxidizer indicates that 53.0 v% of N_2_ in the oxidizer is required to operate the GP-SIRE in the experiment at maximum power.

### 4.3 Comparison of predicted maximum power for the GP-SIRE operating with the three different bio-syngas mixtures

**Fig. 14:**
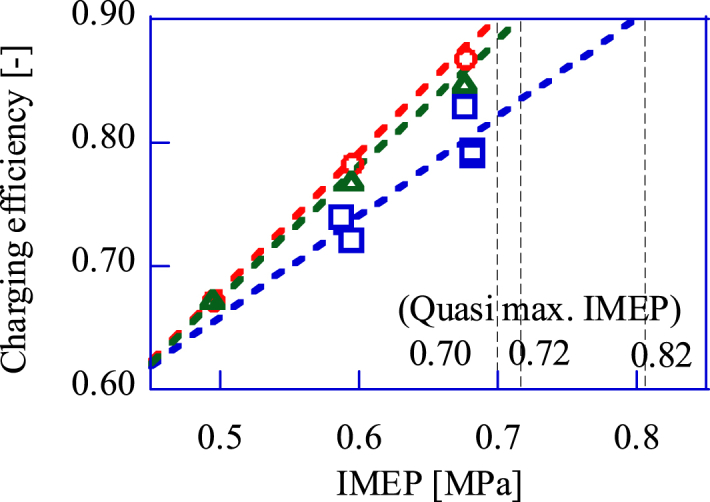
Charging efficiency against IMEP. □:Upgrade bio-syngas, △:Bio-syngas with H_2_, 〇:Bio-syngas with CH_4_.

Fig. 14 shows the relation between charging efficiency and the maximum IMEP for the three different bio-syngas mixtures. The bio-syngas with CH_4_ has a lower power at the maximum charging efficiency. Since The stoichiometric amount of air of H_2_ and CO is about 4 times greater than CH_4_, and that results the heat input reduction. Table 2 shows the fuel's calorific value and the volume fraction of each component. The upgrade bio-syngas has a higher CO fraction and a lower CH_4_ fraction in the fuel than the bio-syngas with H_2_. CO is a component with the higher LHV than H_2_. Therefore, when the upgrade bio-syngas is supplied, the stoichiometric amount of air does not increase excessively and the fuel's LHV in the cylinder increases. Consequently, the maximum IMEP is highest under the upgrade bio-syngas operation.

**Table 2:** The LHV and the volume fraction of chemical species in each gaseous fuel.

	H2 [v%]	CH4 [v%]	CO [v%]	N2 [v%]	CO2 [v%]	LHV [MJ/m3-LHV]	Supplied heat quantity [kW]	H2, CH4, O2 flow rate [L/min]
Bio-syngas without addition	14.2	2.2	17.9	50.8	13.5	4.3	5.8	×
Bio-syngas with H2 cal.	31.4	1.2	15.4	41.8	9.1	5.7	7.1	13.9
Bio-syngas with H2 cal.	43.5	1.0	12.6	34.4	7.5	6.6	8.1	21.1
Bio-syngas with H2 cal.	51.3	0.8	10.9	29.7	6.4	7.2	8.7	25.2
Bio-syngas with CH4 cal.	12.0	5.2	18.8	51.4	11.1	5.7	7.1	2.7
Bio-syngas with CH4 cal.	11.6	7.6	18.4	50.1	10.9	6.6	8.1	4.4
Bio-syngas with CH4 cal.	11.5	8.6	18.2	49.6	10.7	7.0	8.4	5.0
Upgrade bio-syngas	13.3	2.0	28.5	43.2	11.5	5.7	7.1	4.7
Upgrade bio-syngas	15.4	2.2	33.3	36.3	11.3	6.6	8.1	8.8
Upgrade bio-syngas cal.	17.9	2.4	39.0	28.2	11.0	7.9	9.3	13.3

### 4.4 Calculation of additive gas cost under maximum power conditions

The maximum IMEP was the highest for the upgrade bio-syngas operation. However, the gas additives required for these operating conditions are not always the least expensive. Therefore, to calculate the cost-effectiveness of the upgrade bio-syngas, the flow rates of the three additive gases required for maximum power operating conditions are determined, and the cost of each additive gas is calculated.

1) Three types of additive gases' flow rate

The flow rates of combustible gases, required for maximum power operation of the GP-SIRE, are predicted as follows: H_2_ and CH_4_ flow rates are obtained from the heat input and fuel composition for the expected maximum power operation in Table 2. H_2_ flow rate is 25.2 L/min and CH_4_ is 5.0 L/min. The flow rate of O_2-quasi_ for the upgrade bio-syngas is determined from the heat input required for the expected maximum power, by a linear relation between the fuel's LHV and N_2_ or O_2_ ratio supplied to the gasifier (see Fig. 13). As a result, the flow rate of O_2-quasi_ is 13.3 L/min.

2) Cost calculations for additive gases

The procedure for calculating the cost of O_2_, H_2_, or CH_4_ for supplemental use in woody biomass power generation with a small GP-SIRE is as follows: 1) focus on the CAPEX of the power generation equipment and the CAPEX of the equipment for gas generation; 2) use electricity generated by renewable energy required to produce O_2_, H_2_, or CH_4_. The most promising candidates for renewable energy are photo voltaic (PV) and wind power. In both cases, CO_2_ emissions during the gas production and the “fuel costs” for the power generation are negligible. Therefore, the production cost of O_2_, H_2_, and CH_4_ is "power generation equipment cost“ + ”equipment cost” for gas production.

O_2_ can be produced by electrolysis of H_2_O or by pressure swing adsorption (PSA) by air as a raw material. Generally, the production of O_2_ from H_2_O by electrolysis has a higher production intensity [kWh/Nm^3^-O_2_] than the PSA method. The O_2_PVSA method is for inexpensive O_2_ production, with the cost of 9.8 JPY/Nm^3^ [21]. The PV or wind power generation costs in Japan are expected to be around 5.8 JPY/kWh for PV and 6.6 JPY/kWh for wind by 2030, respectively [22]. Since O_2_ production requires 0.34 kW/Nm^3^ [21], the total cost is 12.18 yen/Nm^3^-O_2_. H_2_ is mainly produced by electrolysis of H_2_O. Shibata et al. estimated that the H_2_ production cost would be 37 JPY/Nm^3^-H_2_ when the unit cost of electricity from renewable energy is 7.0 JPY/kWh [23]. CH_4_, which is called synthetic methane with CO_2_ produced by methane fermentation and H_2_ produced by electrolysis of H_2_O, is attracting attention as a carbon-neutral one. In this case, the production cost is 140 JPY/Nm^3^-CH_4_ [23]. Part of the CH_4_ produced in methane fermentation is supplied as city gas. Although the basic unit cost of CNG is 94.91 JPY/Nm^3^, the CNG is not carbon-neutral. The effect of hydrogen investigated by Shivapuji shows good results [24]. This report shows not only hydrogen effect but also “inert gas” effect. Though low price hydrogen will be produced via low price photo voltaic, oxygen from air will be produced at the same time. The both species should be used effectively.

3) The upgrade bio-syngas usefulness from a cost perspective

Table 3 shows, for each gaseous fuel, the required flow rate of additive fuel or oxidizer O_2-quasi_ and the required cost for maximum power operation of the GP-SIRE. The required O_2-quasi_ flow rate shown in Table 3 differs from the required O_2-quasi_ flow rate shown in Table 2 since the O_2_ concentration obtained from the PSA is calculated as 0.88 in Table 3. Therefore, the least expensive case is the case operated with the upgrade bio-syngas. This means that the usefulness of the upgrade bio-syngas from a cost standpoint is demonstrated.

**Table 3:** Flow rate of added fuel and oxidizer required for maximum power operation and required cost for each gaseous fuel.

Gaseous fuel	Added fuel	Production process	Oxidizer for gasification	O2-quasi, H2, CH4 flow rate [L/min]	Carbon dioxide emission [g/kWh]	Additional [JPY/h]	Max. IMEP of SI-ICE [MPa]	Efficiency of SI-ICE [−]	Exhaust gas from SI-ICE		
									HC [vppm]	CO [v%]	NOx [vppm]
Bio-syngas	x	Gasification	Air	×	Caron neutral	×	0.58	0.41	×	0.06	223
Upgrade bio-syngas	x	Gasification	Air + O2-quasi	15.5	Caron neutral	11.33	0.82	0.46	59	0.04	1774
Bio-syngas with	H2	Electrolysis	Air	25.2	Caron neutral	55.94	0.72	0.43	77	0.01	1512
Bio-syngas with	CH4	Natural gas	Air	5.0	70.2	28.47	0.70	0.44	11	0.04	627
Bio-syngas with	CH4	Methanation	Air	5.0	Carbon neutral	42.00	0.70	0.44	11	0.04	627
13A (Ref.)	×	Natural gas	×	21.2	171.3	120.73	0.90	0.35	142	0.05	2408

## 5 Conclusions

In this experiment, the three different bio-syngas mixtures were tested to determine the most cost-effective fuel for a small SIRE. The engine performance was evaluated under maximum power operating conditions with the IMEP as the evaluation index, and the fuel costs were estimated by determining the flow rate of oxidizer or added fuel in each mixture. As a result, the following facts were found.

(1) The predicted IMEP is highest in case of the upgrade bio-syngas operating. The supply of the upgrade bio-syngas was able to suppress the excessive increase in a stoichiometric amount of air with increasing LHV simultaneously.
(2) A comparison of gas production costs, calculated from the oxidizer O_2-quasi_ flow rate, H_2_ flow rate, and CH_4_ flow rate in the three fuels, revealed that the upgrade bio-syngas with O_2_ is the least expensive. In other words, the upgrade bio-syngas is the most advantageous in terms of fuel cost at maximum engine power.

## CRediT authorship contribution statement

**Hiroshi Enomoto:** Writing – original draft, Software, Resources, Project administration, Methodology, Funding acquisition, Formal analysis, Data curation, Conceptualization. **Ryo Nakagawa:** Writing – original draft, Visualization, Validation, Data curation. **Ayako Yoshimichi:** Writing – review & editing, Validation.

## Declaration of competing interest

The authors declare that they have no known competing financial interests or personal relationships that could have appeared to influence the work reported in this paper.
