# Supplementary material for: Economic analysis of reciprocating engine generating with bio-syngas at predicted maximum power condition
Source: Heliyon. 2024 Jul 11;10(15):e34338. doi: 10.1016/j.heliyon.2024.e34338 (PMC11320148; doi:10.1016/j.heliyon.2024.e34338)
Supplement: Multimedia component 1 [file mmc1.docx]

Supplement

Lists of the physical properties in this paper are shown in Table S- 1. The symbol “*i*” represents the chemical species H_2_, N_2_, O_2_, CH_4_, CO, CO_2_, H_2_O.

§1 List of Symbols

Table S- 1 Physical properties, 13A is city gas.

*Bo* [m] Bore

COV_IMEP_ [Pa] Indicated mean effective pressure

*CR* [-] Compression Ratio

EAR [-] Excess Air Ratio

*e_i_* [-] Error of element *i*

*e*_m_ [-] Error of calculation result

IMEP_avg_ [Pa] Average IMEP over the measurement period

IMEP*_n_* [Pa] IMEP *n*th cycle IMEP Low Heating Value of chemical species *i* (*i* = H_2_, CH_4_, CO)

LHV_fuel_ [MJ/m^3^] Low Heating Value of fuel

LHV*_i_*　　　　　　[MJ/m^3^] Low Heating Value of chemical species *i* (*i* = H_2_, CH_4_, CO)

LHV_mixed fuel_ [MJ/m^3^] Low Heating Value of mixed fuel

*Lc* [m] Connecting rod length

*l*o　 [m] Piston offset

*MW_i_* [kg/mol] Molecular weight of chemical species *i*

*M_i_*_.ICE.init_ [kg] Mass of chemical species *i* in cylinder before combustion

*M_i_*_.ICE.θ_ [kg] Mass of chemical species *i* in cylinder at crank angle θ

*M*_i kWh_  [kg/kWh] Mass of chemical species *i* consumed per kWh

*M*_mix per cycle_ [kg] Mass of gas in cylinder per cycle

*mol_i._*_ICE.init_ [mol] Number of moles of chemical species *i* in cylinder before combustion

*mol_i._*_ICE.θ_ [mol] Number of moles of chemical species *i* in the cylinder at crank angle θ

*mol*_i kWh_ [mol] Molecular weight of chemical species *i* consumed per kWh

(*i* = CO, CO_2_, CH_4_, H_2_, H_2_O)

*N*_cycle_ [-] Number of cycle

*p_θ_* [Pa] In-cylinder pressure at crank angle θ

*p_θ.avg_* [Pa] Average in-cylinder pressure at crank angle θ ?

*Q*_HRR.max_ [J] Maximum cumulative heat release rate

*Q*_HRR_*_.θ.avg_*  [J] Average cumulative heat release rate up to crank angle θ

*Q*_input_ [W] Heat to be supplied per unit time

*Q*_input per cycle_ [J] Heat to be supplied per cycle

*Q*_output_  [J] Shaft power

*RR_θ.avg_* [-] Average reaction rate up to crank angle *θ*

*Ri* [kJ/kg/K] Gas constant of chemical species *i*

*R_θ_* [kJ/kg/K] Gas constant of in-cylinder at crank angle θ

*rV_i_*_.GC_  [-] Volume fraction of chemical species *i* measured by gas chromatography

(*i* =H_2_, N_2_, O_2_, CH_4_, CO, CO_2_)

*rV_i_*_.GC.mod_ [-] Volume fraction of chemical species *i* in bio-syngas

(*i* =H_2_, N_2_, CH_4_, CO, CO_2_)

*rV_i_*_.ICE.init_ [-] In-cylinder volume fraction of chemical species *i* before combustion starts

(*i* =H_2_, N_2_, CH_4_, CO, CO_2_)

*rV_i_*_.fuel_ [-] Volume fraction of chemical species *i* in fuel

(*i* =H_2_, N_2_, CH_4_, CO,CO_2_)

*S*_ICE_ [rpm] Engine speed

*T*_θ_ [K] In-cylinder temperature at crank angle θ

*t*_ig_　 [deg] Ignition timing

*V*_air.G.inlet_  [L/min] Flow rate of air for the gasifier intake

*V*_air.ICE.inlet_ [L/min] Flow rate of air which intake by engine

*V*_air._*_i._*_th_　　　　　 [m^3^/m^3^] Stoichiometric amount of air ratio for chemical species *i* (*i* = H_2_, CH_4_, CO)

*V*_air.suction_ [L/min] Aspirated air flow rate except from the air inlet at gasifier

*V*_disp_ [m^3^] Displacement flow rate

*V*_fuel_ [L/min] Fuel flow rate

*V_i_* [L/min] Flow rate of chemical species *i*

(*i* =H_2_, N_2_, CH_4_, CO,CO_2_)

*V*_i kWh_ [L/kWh] Volume of gas *i* per kWh (ICE shaft power) supplied to ICE

*V*_mix_ [L/min] Flow rate of mixture

*V*_mix per cycle_ [L] Volume of mixture to be supplied per cycle

*V*_st_ [m^3^] Stroke volume

*V*_syn_ [L/min] Bio-syngas flow rate

*V*_syn.mod_ 　　　　[L/min] Modified bio-syngas flow rate

*V_θ_* [m^3^] In-cylinder volume at crank angle *θ*

*v_i_* [-] Measurement result of element *i*

*v*_m_ [-] Value of calculation results

*W*_Otto.avg_ [J] Average value of Otto cycle during the measurement period

*W*_indicated._ [J] Value of indicated work during the measurement period

*W*_indicated.avg_ [J] Average value of indicated work during the measurement period

*W*_pump.avg_ [J] Average value of pump losses during the measurement period

*Y*_CH4 price_ [JPY/Nm^3^] CHG-derived CH_4_ sales price

*Y*_i,cost_ [JPY/Nm^3^] Production cost per unit volume of gas *i*

(*i* =H_2_, CH_4_, CO)

*Y*_require cost_ [JPY/hour] Cost of gas consumed by ICE operation per hour

*η*_charge_ [-] Charging efficiency

*η*_indicated_ [-] Indicated thermal efficiency

*θ* [deg] Crank angle

*θ*_combustion.end_　　　[deg] Crank angle of combustion end

§2 Calculation of each variable for the gasifier

The calculation method for the indirect values are shown below.

§§2.1 The bio-syngas flow rate and the composition

Assuming that the mass ratio of N_2_ in the pellets is very small compared to the mass ratio of N_2_ in the air, and that N_2_ does not react during the gasification process, it should be considered that the mass of N_2_ in the air supplied from the gasifier inlet and aspirated by the gasifier from the later parts of the reactor will be equal to the mass of N_2_ in the bio-syngas after gasification. Consequently, it is written as S-1.

$$\begin{aligned} 0.79\left( V_{air.G.inlet}+V_{air.suction} \right)=V_{syn}rV_{N_{2}.GC}\#S-1 \end{aligned}$$

Assuming that all O_2_ in the air supplied from the gasifier inlet is consumed in the gasification process, the volume ratio of O_2_ measured by gas chromatography is due to O_2_ in the air inlet from the reactor and later parts, so it is written as S-2.

$$\begin{aligned} V_{air.suction}=V_{syn}\frac{rV_{O_{2}.GC}}{0.21}\#S-2 \end{aligned}$$

*V*_syn_ is calculated by substituting S-2 into S-1 to obtain Equation S-3.

$$\begin{aligned} V_{syn}=\frac{0.79V_{air.G.inlet}}{\left( rV_{N2.GC}-\frac{0.79}{0.21}rV_{O_{2}.GC} \right)}\#S-3 \end{aligned}$$

The inside of the reactor in this paper was under negative pressure, so the air was sometimes aspirated at the joints. Thus, the following equation is to modify the intake air flow rate．

$$\begin{aligned} V_{syn.mod}=V_{syn}-V_{air.suction}\#S-4 \end{aligned}$$

H_2_O in the bio-syngas is removed when measured by gas chromatography. The following modified equation is for the syngas composition supplied to the engine, considering the aspirated air and the removed H_2_O.

$$\begin{aligned} rV_{i.GC.mod}=\frac{V_{syn}}{V_{syn.mod}}\left( 1-rV_{H_{2}O.GC.mod} \right)rV_{i.GC} ,\#S- \end{aligned}5$$

for *i* = H_2_, CH_4_, CO, CO_2_．

$$\begin{aligned} rV_{N_{2}.GC.mod}=\frac{V_{syn}\left( 1.00-F_{H_{2}O.GC.mod} \right)rV_{N_{2}.GC}-0.79V_{air.suction}}{V_{syn.mod}} \#S-6 \end{aligned}$$

*rV*_H2O.GC.mod_ was between 1.0% and 2.0%, so it is determined as 1.5%.

§3 Combustion analysis of SI-ICE

§§3.1 Fuel flow rate to input into the engine and the composition

The bio-syngas added with H_2_, CH_4_, and CO_2_ are for the fuel in this experiment. Therefore, the fuel flow rate to be input to the engine is as follows.

$$\begin{aligned} V_{fuel}=V_{syn.mod}+V_{{CH}_{4}}+V_{H_{2}}+V_{{CO}_{2}} \#S-7 \end{aligned}$$

The gas composition in the fuel is calculated by S-6.

$$\begin{aligned} rV_{i.fuel}=\frac{V_{syn.mod}rV_{i.GC .mod}+V_{i}}{V_{fuel}} \#S-8 \end{aligned}$$

for *i* = H_2_, CH_4,_ CO_2_

$$\begin{aligned} rV_{i.fuel}=\frac{V_{syn.mod}rV_{i.GC .mod}}{V_{fuel}} \#S-9 \end{aligned}$$

for *i* = N_2_, CO, H_2_O

§§3.2 Excess air ratio

The excess air ratio is defined as the ratio of the actual air intake into the engine to the amount of air required for complete combustion of the fuel (stoichiometric amount of air). The stoichiometric amount of air for each gas species can be obtained by considering the chemical reaction equation for their complete combustion. Through the stoichiometric amount of air and the volume fraction of each gas species, the stoichiometric amount of air for a fuel containing multiple gas species can be calculated by S-10.

$$\begin{aligned} V_{air.syn.th}=rV_{H_{2}.GC.mod}V_{air.H_{2}.th}+rV_{{CH}_{4}.GC.mod}V_{air.{CH}_{4}.th}+rV_{CO.GC.mod}+V_{air.CO.th} \#S-10 \end{aligned}$$

The stoichiometric amount of air of the fuel, added with H_2_, CH_4_, and CO_2_ to the bio-syngas, is calculated by S-11.

$$\begin{aligned} V_{air.mix.th}=\frac{V_{syn.mod}V_{air.syn.th}+{V_{{CH}_{4}}V}_{air.{CH}_{4}.th}+V_{H_{2}}V_{air.H_{2}.th}}{V_{fuel}} \#S-11 \end{aligned}$$

Considering that there are two types of air intake to the engine: outside air which is intake directly from the engine inlet and air contained in the fuel lines, the EAR is calculated as S-12

$$\begin{aligned} EAR=\frac{V_{air.suction}{+V}_{air.ENG.intake}}{V_{air.mix.th}V_{fuel}} \#S-12 \end{aligned}$$

§§3.3 The LHV of the fuel and the heat input

The LHV is a physical property that can be calculated from the enthalpy change of the combustion reaction of the flammable gas. Therefore, the LHV of the fuels that are input to the engine is calculated as S-13 by the LHV of each gas species and its volume fraction in the fuel.

$$\begin{aligned} {LHV}_{mixed fuel}=\sum\left( rV_{i.fuel}{LHV}_{i} \right) \#S-13 \end{aligned}$$

for *i* = H_2_, CH_4_, CO．

By multiplying the fuel’s LHV [MJ/m^3^] in S-13 by the fuel flow rate, the heat per unit time input to the engine can be calculated as S-14.

$$\begin{aligned} Q_{input}=V_{fuel}{\frac{{10}^{3}}{60}b}_{fuel}\#S-14 \end{aligned}$$

The engine intakes the air mixture only once per cycle. Since the engine in this experiment is a four-stroke engine, the crankshaft makes two revolutions per cycle. Therefore, the heat input per cycle can be calculated as S-15.

$$\begin{aligned} Q_{input per cycle}=V_{fuel}\frac{{10}^{3}}{60}{LHV}_{fuel}\frac{2\times60}{S_{ICE}} \# \end{aligned}$$

$$\begin{aligned} =2\times{10}^{3}\frac{Q_{input}}{S_{ICE}} \#S-15 \end{aligned}$$

§§3.4 In-cylinder volume

The ICE in this experiment has a piston offset. The engine piston displacement and an in-cylinder volume can be expressed by a crank angle as follows.

$$\begin{aligned} Z_{\theta}=\sqrt{\left( \frac{St}{2}+L_{c} \right)^{2}-{l_{o}}^{2}}-\frac{St}{2}\cos\varphi-\sqrt{{L_{c}}^{2}+\sin^{2} \varphi}\#S-16 \end{aligned}$$

$$\begin{aligned} \varphi=\frac{\pi}{180}\theta+\sin^{-1} \left( \frac{l_{o}}{L_{c}+\frac{St}{2}} \right)\#S-17 \end{aligned}$$

$$\begin{aligned} V_{\theta}=\frac{\pi{B_{o}}^{2}}{4}Z_{\theta}+\frac{V_{disp}}{CR-1} \#S-18 \end{aligned}$$

§§3.5 Heat generation rate

Assuming that the in-cylinder charge is an ideal gas and there is no leakage from the in-cylinder, the heat that is released into the in-cylinder by combustion is S-19 by the first law of thermodynamics.

$$\begin{aligned} dQ=d\left( {mC}_{v}T \right)+pdV \#S-19 \end{aligned}$$

The equation of state and Meyer’s relation obtains S-20.

$$\begin{aligned} dQ=\frac{Vdp+\kappa pdV}{\kappa-1}-\frac{pVd\kappa}{\left( \kappa-1 \right)^{2}} \#S-20 \end{aligned}$$

The differential formula per crank angle is as follows.

$$\begin{aligned} \frac{dQ}{d\theta}=\frac{1}{\kappa-1}\left( V_{\theta}\frac{dp}{d\theta}+\kappa p\frac{dV_{\theta}}{d\theta} \right)-\frac{pV_{\theta}}{\left( \kappa-1 \right)^{2}}\frac{d\kappa}{d\theta}\#S-21 \end{aligned}$$

For simplicity, the specific heat ratio is assumed constant when considering the heat generation rate, and the obtained cylinder pressure is the average of 150 cycles, so the heat generation rate is as S-22.

$$\begin{aligned} {\frac{dQ}{d\theta}}_{avg}=\frac{V_{\theta}\frac{{dP}_{\theta}}{d\theta}+\kappa P_{\theta}\frac{{dV}_{\theta}}{d\theta}}{\kappa-1} \#S-22 \end{aligned}$$

§§3.6 Reaction rate

As in S-23, the heat generation rate from the combustion start time (ignition timing) to any crank angle is considered to have a value obtained by integrating the crank angle.

$$\begin{aligned} Q_{HHR.\theta.avg}=\int{\frac{dQ}{d\theta}}_{avg} d\theta\#S-23 \end{aligned}$$

from *θ* = *t*_ig_ to *θ*

*Q*_HRR.max_ represents the accumulated calorific value generated by combustion to the end of combustion position θ_combustion.end_. The reaction rate *RR_θ_*_.avg_ to the crank angle θ is defined by the following equation.

$$\begin{aligned} {RR}_{\theta.avg}=\frac{Q_{HHR.\theta.avg}}{Q_{HRR.max}} \#S-24 \end{aligned}$$

S-23 shows the generated calorific value to a given crank angle in the total calorific value by the combustion. In other words, it represents the ratio of the combustion reaction progress.

§§3.7 In-cylinder temperature

The following section describes the procedures to estimate the in-cylinder temperature.

The flow rate of the air mixture input to the engine is determined by S-25.

$$\begin{aligned} V_{mix}=V_{syn.mod}+V_{air.suction}+V_{13A}+V_{H_{2}}+V_{N_{2}}+V_{{CO}_{2}}+V_{air.ICE.inlet} \#S-25 \end{aligned}$$

The chemical species composition of the mixture is determined by the following equation.

$$\begin{aligned} {rV}_{i.ICE.init}=\frac{V_{i}+{rV}_{i.fuel}V_{fuel}}{V_{mix}} \#S-26 \end{aligned}$$

for *i* = H_2_, CO_2_, CH_4_．

$$\begin{aligned} {rV}_{i.ICE.init}=\frac{{rV}_{i.fuel}V_{fuel}}{V_{mix}} \#S-27 \end{aligned}$$

for *i* = CO, H_2_O．

$$\begin{aligned} {rV}_{N_{2}.ICE.init}=\frac{0.79\left( V_{air.ICE.inlet}+V_{air.suction} \right)+{rV}_{N_{2}.fuel} V_{fuel}}{V_{mix}} \#S-28 \end{aligned}$$

$$\begin{aligned} {rV}_{O_{2}.ICE.init}=\frac{0.21\left( V_{air.ICE.inlet}+V_{air.suction} \right)}{V_{mix}} \#S-29 \end{aligned}$$

The flow rate of the mixture is recalculated by S-30 to the volume of mixture input to the engine per cycle.

$$\begin{aligned} V_{mix per cycle}=\frac{2V_{mix}}{S_{ICE}} \#S-30 \end{aligned}$$

The mass of each gas species in the mixture input to the cylinder is determined by S-31.

$$\begin{aligned} M_{i.ICE.init}=V_{mix per cycle}{{10}^{-3}\rho}_{i} {rV}_{i.ICE.init} \#S-31 \end{aligned}$$

for *i* = H_2_, N_2,_ O_2_, CH_4_, CO, CO_2_, H_2_O．

The mass of each gas species in S-31 is to determine the number of moles of each gas species by S-32.

$$\begin{aligned} {mol}_{i.ICE.init}=\frac{M_{i.ICE.init}}{{MW}_{i}} \#S-32 \end{aligned}$$

for *i* = H_2_, N_2_, O_2_, CH_4_, CO, CO_2_, H_2_O．

The gas composition of in-cylinder changes as combustion proceeds. Reaction kinetics has recently been used to describe this change history in detail. For simplicity, it is considered that the composition of the in-cylinder as a function of the reaction rate. Therefore, if the composition of the in-cylinder approaches the composition after complete combustion as the reaction rate progresses as S-33 to S-37, the number of moles of the chemical species at a given crank angle is obtained.

$$\begin{aligned} {mol}_{i.ICE.\theta}=\left( 1-{RR}_{\theta.avg} \right){mol}_{i.ICE.init}\#S-33 \end{aligned}$$

for *i* = H_2_, CH_4_, CO．

$$\begin{aligned} {mol}_{N_{2}.ICE.\theta}={mol}_{N_{2}.ICE.init} \#S-34 \end{aligned}$$

$$\begin{aligned} {mol}_{O_{2}.ICE.\theta}={mol}_{O_{2}.ICE.init}-{RR}_{\theta.avg}\left( {0.5mol}_{H_{2}.ICE.init}+{0.5mol}_{CO.ICE.init}+{2.0mol}_{{CH}_{4}.ICE.init} \right)\#S-35 \end{aligned}$$

$$\begin{aligned} {mol}_{{CO}_{2}.ICE.\theta}={mol}_{{CO}_{2}.ICE.init}+{RR}_{\theta.avg}\left( {mol}_{CO.ICE.init}+{mol}_{{CH}_{4}.ICE.init} \right)\#S-36 \end{aligned}$$

$$\begin{aligned} {mol}_{H_{2}O.ICE.\theta}={mol}_{H_{2}O.ICE.init}-{RR}_{\theta.avg}\left( {mol}_{H_{2}.ICE.init}+{2.0mol}_{{CH}_{4}.ICE.init} \right).\#S-37 \end{aligned}$$

As in S-38, the mass of each gas species at a given crank angle is obtained by multiplying the number of moles in S-33 through S-37 by the molecular weight.

$$\begin{aligned} M_{i.ICE.\theta}={MW}_{i} {mol}_{i.ICE.\theta} \#S-38 \end{aligned}$$

for *i* = H_2_, N_2_, O_2_, CH_4_, CO, CO_2_, H_2_O．

The mass of each gas species is added together to obtain the in-cylinder gas mass, as in S-39.

$$\begin{aligned} M_{mix per cycle}= \sum M_{i.ICE.init}=\sum M_{i.ICE.\theta}\#S-39 \end{aligned}$$

The mass fraction of each gas species at a given crank angle, as in S-40, is determined.

$$\begin{aligned} {rM}_{i.ICE.\theta}= \frac{M_{i.ICE.\theta}}{M_{mix per cycle}} \#S-40 \end{aligned}$$

for *i* = H_2_, N_2_, O_2_, CH_4,_ CO, CO_2_, H_2_O．

The average gas constant of the in-cylinder gas at a given crank angle is determined as S-41.

$$\begin{aligned} R_{\theta}=\sum R_{i} {rM}_{i.ICE.\theta}\#S-41 \end{aligned}$$

By substituting the mass of gas and gas constant in the cylinder by the above procedure into the equation of state, the in-cylinder temperature is estimated as S-42.

$$\begin{aligned} T_{\theta}=p_{\theta.avg}\frac{M_{mix per cycle}}{R_{\theta}} \#S-42 \end{aligned}$$

§§3.8 IMEP

From the cylinder pressure in the experimental result, a finger pressure diagram is drawn. The value by integrating the pressure diagram with rightward rotation as positive is called the indicated work. Among this, the work performed in the compression and expansion processes is defined as compression and expansion work. The work required for the exhaust and intake processes is defined as pump loss. They are represented as S-43 and S-44, respectively. *N*_cycle_ = 150.

$$\begin{aligned} W_{Otto.avg}=\frac{{\sum\left( p_{\theta.ave}\times{dV}_{\theta} \right)}_{exp}-\sum\left( p_{\theta.ave}\times{dV}_{\theta} \right)_{comp}}{N_{cycle}} \#S-43 \end{aligned}$$

exp: from TDC to 179deg-ATDC，

comp: from 180deg-BTDC to TDC，

$$\begin{aligned} W_{pump.avg}=\frac{\sum\left( p_{\theta.ave}{dV}_{\theta} \right)_{exp}-\sum\left( p_{\theta.ave}{dV}_{\theta} \right)_{comp}}{N_{cycle}} \#\#S-44 \end{aligned}$$

exp: from 180edg-ATDC to 359deg-ATDC，

comp: from 360deg-BTDC to 181deg-BTDC．

The indicated work is shown in S-45.

$$\begin{aligned} W_{indicated.avg}=W_{Otto.avg}- W_{pump.avg} \#S-45 \end{aligned}$$

The IMEP is defined as a value by dividing the indicated work by the process volume, so the IMEP is shown in S-46.

$$\begin{aligned} {IMEP}_{avg}=\frac{W_{Otto.avg}-W_{pump.avg}}{V_{disp}} \#S-46 \end{aligned}$$

Since the cylinder pressure is the average of 150 cycles, each index is also the average of 150 cycles.

The variation rate of the IMEP is calculated by S-47.

$$\begin{aligned} {COV}_{IMEP}= \frac{\sqrt{\sum\frac{\left( {IMEP}_{n}-{IMEP}_{avg} \right)^{2}}{N_{cycle}-1}}}{{IMEP}_{avg}}\#S-47 \end{aligned}$$

The indicated efficiency is calculated by S-48.

$$\begin{aligned} \eta_{indicated}=\frac{W_{indicated}}{Q_{input per cycle}} \#S-48 \end{aligned}$$

§§3.9 Gas cost calculation

The cost, corresponding to one hour of operation of the GP-SIRE is obtained from the O_2_ flow rate when O_2_ is added to the gasifier oxidizer. The same calculation is performed for the case of adding H_2_ and CH_4_ to the bio-syngas.

$$\begin{aligned} Y_{required cost}=Y_{i cost} \times V_{i} \#s-49 \end{aligned}$$

for *i* = H_2_, O_2_, CH_4_

When city gas (LHG) is used as CH_4_, the following calculation is made from the sales price of city gas.

$$\begin{aligned} Y_{required cost}=Y_{CH4 price} \times V_{i} \#s-50 \end{aligned}$$

§§3.10 CO_2_ emission calculation

When CH_4_ derived from the fossil fuel is combusted, the CO_2_ emitted per kWh is calculated as follows. The absolute value of the energy in 1 kWh of electricity is 3.6 MJ.

From the following equation, the required CH4 flow rate is calculated.

$$\begin{aligned} V_{CH4 kWh}=\frac{3.6 \times{10}^{6}}{Q_{\mathrm{input}}} \times\frac{1}{60} \times V_{{CH}_{4}}\#s-51 \end{aligned}$$

The molecular weight of CH_4_ is determined by the following equation, and the mass of CO_2_ is calculated assuming the complete combustion of CH_4_.

$$\begin{aligned} {mol}_{{CH}_{4}\mathrm{kWh}}=\frac{V_{i kWh}}{22.4}\#s-52 \end{aligned}$$

$$\begin{aligned} M_{{CO}_{2}\mathrm{kWh}}={mol}_{{CH}_{4}\mathrm{kWh}} \times M_{w {CH}_{4}}\#s-53 \end{aligned}$$

§§3.11 Charging efficiency

The ratio of the gas volume intake to the maximum gas volume that a GP-SERE can intake is determined by the following calculation.

$$\begin{aligned} \eta_{\mathrm{charge}}=\frac{V_{\mathrm{mix}}}{V_{\mathrm{st}}\times{10}^{3} \times\frac{S_{\mathrm{ICE}}}{2}} \#s-54 \end{aligned}$$

§4 Accuracy

Table S- 2 shows the measurement range, accuracy, and sensitivity of the measurement equipment in the experiment. Almost all variables are indirect measurements of the measured value, so the errors propagate. The following equations are for calculating error propagation.

In the case that the equation is expressed in terms of additions and subtractions, such as *vm* = *v*1 + *v*2 + *v*3 +…,

$$\begin{aligned} e_{m}=\sum e_{i} \#S-55 \end{aligned}$$

In the case that the formula is expressed by multiplication and division as *v*_m_ = *v*_1_×*v*_2_×*v*_3_×…,

$$\begin{aligned} e_{m}=\sqrt{\sum\left( e_{i} \right)^{2}} \#S-56 \end{aligned}$$

Error propagation calculations for each equation by S-55 and S-56 resulted in a relative error of approximately. The in-cylinder temperature, by the accuracy in Table S- 2, resulted in a relative error of approximately 40%. This error makes it difficult to discuss the in-cylinder temperatures. The in-cylinder temperatures were calculated by the accuracy in Table S- 2, and the relative error was within 1%. Sensitivity shows a reliable amount of change and differs from the accuracy. Therefore, each variable except the in-cylinder temperature is determined to have a relative error of approximately 10%, and the in-cylinder temperature has a relative error of 1% for the change amount although the accuracy of the absolute value is low, in this paper.
